# A review and outlook on visual analytics for uncertainties in functional magnetic resonance imaging

**DOI:** 10.1186/s40708-018-0083-0

**Published:** 2018-07-03

**Authors:** Michael de Ridder, Karsten Klein, Jinman Kim

**Affiliations:** 1 Biomedical and Multimedia Information Technology Research Group, University of Sydney, Sydney, Australia; 2 Department of Computer and Information Science, Universität Konstanz, Konstanz, Germany

**Keywords:** Functional magnetic resonance imaging (fMRI), Visualisation analysis, Uncertainty analysis, Uncertainty visual analytics, Functional connectivity, Issue management

## Abstract

Analysis of functional magnetic resonance imaging (fMRI) plays a pivotal role in uncovering an understanding of the brain. fMRI data contain both spatial volume and temporal signal information, which provide a depiction of brain activity. The analysis pipeline, however, is hampered by numerous uncertainties in many of the steps; often seen as one of the last hurdles for the domain. In this review, we categorise fMRI research into three pipeline phases: (i) image acquisition and processing; (ii) image analysis; and (iii) visualisation and human interpretation, to explore the uncertainties that arise in each phase, including the compound effects due to the inter-dependence of steps. Attempts at mitigating uncertainties rely on providing interactive visual analytics that aid users in understanding the effects of the uncertainties and adjusting their analyses. This impetus for visual analytics comes in light of considerable research investigating uncertainty throughout the pipeline. However, to the best of our knowledge, there is yet to be a comprehensive review on the importance and utility of uncertainty visual analytics (UVA) in addressing fMRI concerns, which we term fMRI-UVA. Such techniques have been broadly implemented in related biomedical fields, and its potential for fMRI has recently been explored; however, these attempts are limited in their scope and utility, primarily focussing on addressing small parts of single pipeline phases. Our comprehensive review of the fMRI uncertainties from the perspective of visual analytics addresses the three identified phases in the pipeline. We also discuss the two interrelated approaches for future research opportunities for fMRI-UVA.

## Introduction

Functional magnetic resonance imaging (fMRI) is a medical imaging modality that measures brain activity via associated blood oxygenation in a series of temporal scans [1, 2]. The resulting four-dimensional (4D) data have three spatial dimensions to represent the brain and one temporal dimension for the functional activity. The modality has played a pivotal role in a number of findings, such as defining a baseline default mode network [3] which can be used as a marker for Alzheimer’s disease [4], and distinguishing features of brains with mental disorders, e.g. [5–8]. There are two main modes of fMRI used in research, resting state (rs-fMRI)—which images the brain while the patient is awake, but no mental task is being performed—and task-based (t-fMRI)—which images the brain while a task is being performed, such as listening to music or answering questions; the mode commonly includes resting periods between task events to provide a baseline comparison [1].

**Fig. 1:**
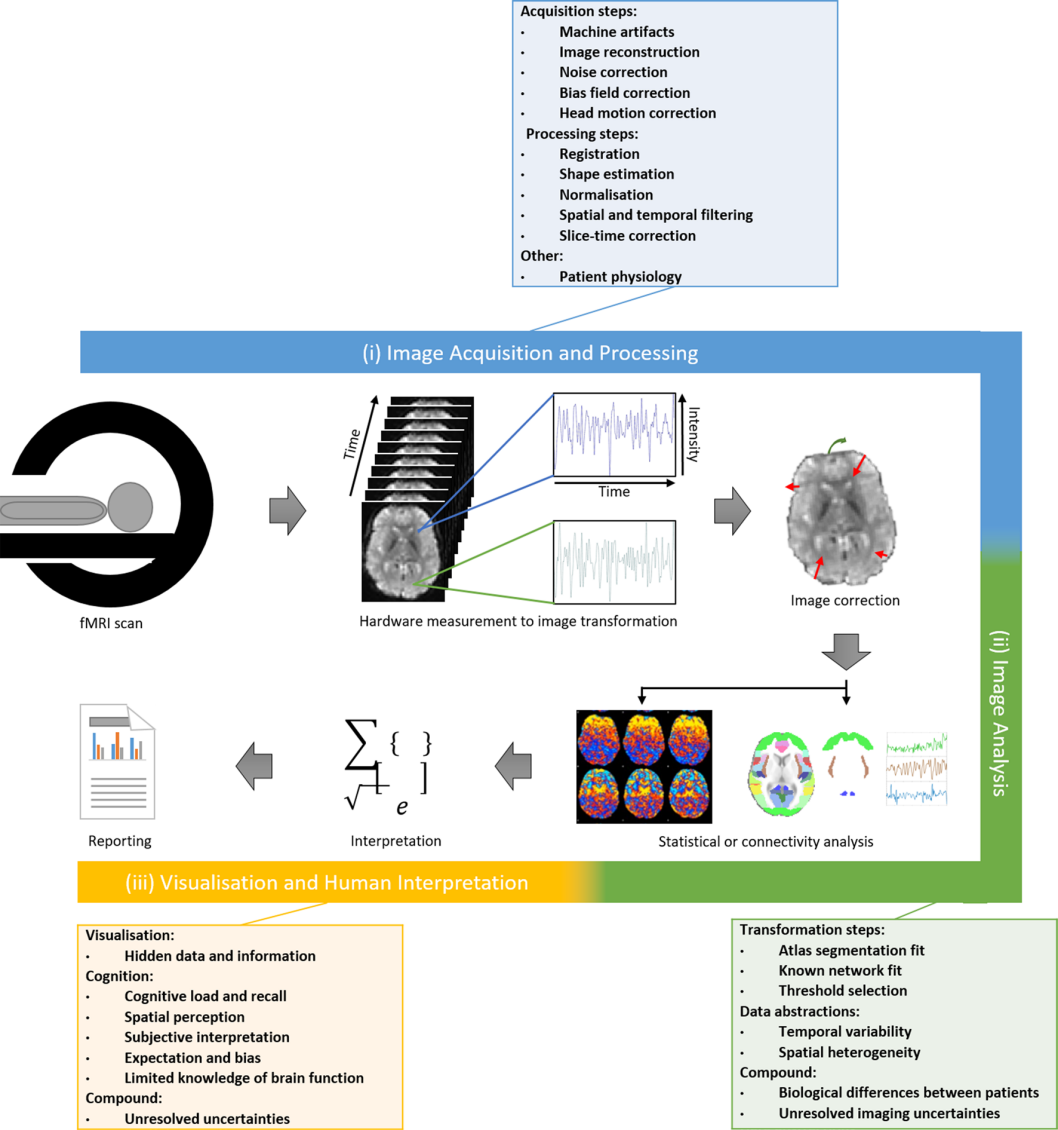
Summary of the fMRI image analysis pipeline, categorised into three phases: (i) image acquisition and processing; (ii) image analysis; and (iii) visualisation and human interpretation. Each of the three phases comprises multiple processing steps that introduce new uncertainties and compound upon existing uncertainties. A summary of the main steps in each phase is denoted in the three boxes

Many of the fMRI discoveries are enabled by advances in the image analysis pipeline, which processes the complex raw 4D fMRI data into meaningful information for interpretation. This pipeline has multiple individual steps that we categorise into three phases, as shown in Fig. 1: (i) image acquisition and processing; (ii) image analysis; and (iii) visualisation and human interpretation. The first phase begins with the scanner taking the images and typically involves processing steps to produce an image from the raw hardware measurements. This usually comprises image reconstruction, motion correction, normalisation, filtering, denoising and registration. The second phase, which is designed to process the fMRI data into meaningful information for interpretation, typically involves statistical image processing methods, such as principal or independent components analysis (PCA/ICA) and voxel clustering—which group the voxels based on temporal signal to determine which areas of the brain are active together and the pattern of this activity. This voxel grouping is different to segmentation and is used, e.g. to categorise and understand diseases. The methods may also be used to assists with improving signal-to-noise ratio (SNR) or in separating matter types in the brain and understanding which parts of the brain are active for t-fMRI, among a range of other uses. These methods are examples of voxel-based functional connectivity analysis. Other methods include seed-based correlation analysis, fractional amplitude of low frequency fluctuations (fALFF) and regional homogeneity analysis. Alternatively, node-based functional connectivity analysis—which maps the 4D fMRI data to 3D spatial regions of interest (ROIs) from a segmented atlas, known as a parcellation—is used to enable the comparison of how similar ROIs are over time and whether these patterns hold across multiple brains, allowing for an understanding of population-level characteristics. These techniques are advantageous in understanding the image data and making it possible to compare one fMRI to another or to a population. More recently, machine learning and deep learning techniques have been applied for many tasks, including image classification to distinguish between diseased and normal fMRI [7, 9, 10]. These techniques rely on insights and labelling from voxel-based and node-based functional connectivity analysis and are thus dependent on their use. The third phase is for human interpretation of the information and communication of the results. This is commonly performed using interactive visualisations [11]. Examples of techniques used in the visualisations include radial views—which list the ROIs as nodes in a circle and display the connections as lines between the nodes—or 3D visualisations of voxel-based results—which use surface and volume renderings of the brain to display which voxels belong to which class, often using different colours [11].

While the fMRI analysis pipeline is used to create valuable information about brain activity, each of the steps in the phases is hampered by a range of uncertainties. Several of these are inherent hardware limitations that arise during the image acquisition and processing phase, such as, low signal-to-noise ratio, low spatial and temporal resolution and questions surrounding how much functional activity the scanners reveal [1]. These are compounded during the image analysis phase, which is contingent on numerous image processing algorithms, each with fundamental weaknesses, in addition to their need for estimation and complex parameter selection. The final phase introduces uncertainty due to information lost in visualising the outcome of the earlier steps, as well uncertainty regarding the human’s ability to correctly understand and interpret the complex data, given the high cognitive load [12–14]. Each of these compound upon the concerns from previous steps due to the inter-dependence of the steps and the flow of the data through the pipeline. A summary of the main steps in each phase that create and compound the uncertainties, derived from [1, 11, 15, 16], is presented in the three outer boxes in Fig. 1.

Efforts have been made to mitigate these uncertainties using interactive visualisation tools, known as visual analytics. Such tools combine human expertise and visual pattern recognition ability with automated processing that enables users to analyse and see the effects of the uncertainties on the data throughout the pipeline. Research has shown that awareness of such issues in the data positively influences human decision making and that it is thus critical to quantify uncertainties for analysis [17, 18]. The push for visual analytics comes as a result of considerable research investigating issues throughout the fMRI analysis pipeline, e.g. [13, 19], and due to the successes of uncertainty visual analytics (UVA) [20, 21] in other related fields. UVA techniques have been broadly explored in related biomedical fields, such as analysis of segmentation errors in medical images including MRI and PET images [22], and widely adopted in a range of other areas [23], such as climate [24], security [25] and astronomy [26]. Reviews have been performed into UVA for specific topics, including medical visualisation [15]—which broadly categorised fMRI within functional imaging, but did not explore the pipeline or visual analytics—promoting the benefits of such research. However, to the best of our understanding, there is yet to be a comprehensive review on the importance of visual analytics in addressing the challenges in fMRI uncertainties. The uncertainties, and issues caused by them, are often recognised as one of the last major hurdles for widespread fMRI use and clinical application. The potential for fMRI to leverage the advances in UVA, which we define as fMRI-UVA, has only started to be explored. For example, there are initial fMRI-UVA approaches, e.g. [27–29], that simplify the presentation of the data to improve human understanding, thereby reducing uncertainty in the interpretation. Similarly, initial attempts have focused on specific aspects of the uncertainty during the processing, primarily towards analysing the temporal sequence, e.g. [30, 31]. These approaches have begun to resolve some of the uncertainties. However, there are numerous others that arise throughout the pipeline, such as threshold selection in the image analysis phase, which have not been targeted. More importantly, despite the progress in addressing the concerns at an individual step or phase level, the more challenging compound effects of the uncertainties, arising from the multiple steps within and among the phases, are not well understood and have received limited attention in research. This greatly impacts fMRI research, raising questions and concerns about many of the findings in the field, such as high false-positive rates in defining clusters of active voxels [12, 14, 32–38]. Consequently, the field of fMRI analysis has not reached its full potential and has seen limited clinical uptake [1, 39]. Thus, fMRI-UVA stands to benefit the field greatly by effectively communicating uncertainties throughout the pipeline.

In this review paper, we first introduce the uncertainties that arise during the fMRI analysis pipeline; in Sect. 2, these are present in both resting-state and task-based studies. Section 3 reviews existing research in image analysis, visualisations and visual analytics, including limitations, the compound effects, and related UVA from other domains. In both these sections, we place specific emphasis on the compound effects of the uncertainties as the data move within and among the phases. We then discuss future directions and opportunities for fMRI-UVA in Sect. 4.

## Uncertainties in fMRI

In this section, we further discuss the range of uncertainties present in the three phases of the fMRI analysis pipeline. These include both data uncertainty, primarily in the first two phases relating to automatic processing: are the data correctly representing the underlying brain function; and human uncertainty, primarily in the third phase, but also in earlier steps, relating to decisions made by humans: is the user correctly interpreting the information and adjusting for the issues arising from the data uncertainty.

### Image acquisition and processing

#### Acquisition steps

Many of the uncertainties in the acquisition steps are derived directly from inherent limitations of the fMRI scanner hardware. A comprehensive review of the fMRI hardware, including its limitations, is beyond the scope of this paper, and interested readers are directed to recent works, e.g. [40–42]. fMRI hardware limitations commonly include poor image resolution, both spatially and temporally; low signal-to-noise ratio; head motion artefacts; functional state differences within the slice sequences in a scan that are caused by the difference in time points taken to get through all the slices sequentially [43]; and others such as the ghosting artefact [44] which results in apparent activation through increased intensity of overlapping signals caused principally by inaccurate timing of reads and magnetic field inhomogeneity [1, 12, 33, 42, 44]. As an example of these limitations, scans typically image the brain at a spatial resolution of 1–2 mm, depending on the hardware and study, with each volumetric scan in the temporal sequence taking between 1 and 3 s, depending on the spatial resolution chosen, resulting in a total scan duration typically taking between 10 and 60 min [42, 45]. However, each cubic millimetre of brain can contain approximately one hundred thousand cells, and the brain can go through tens of state changes per second [42]. This mean an fMRI can only estimate the micro-level function of the brain, likely containing heterogeneity within a voxel, and is susceptible to imaging different brain states as though they are a single contiguous event. While these limitations are inherent to the hardware, some, such as head motion, may be greatly reduced using later processing steps [46].

#### Preprocessing steps

Image processing procedures are used to preprocess fMRI data for analysis. These introduce a range of uncertainties that compound the hardware limitations discussed in Sect. 2.1.1. Required preprocessing steps include motion correction, registration, smoothing and filtering. These steps are common to most medical imaging modalities. However, for fMRI preprocessing, the steps lead to uncertainty as baseline features for each patient, which are used in parameter selection are difficult to determine [47, 48]. For example, landmarks which are often used in motion correction and registration are not easily distinguished in fMRI data [41]. Physiological monitoring, such as head and eye tracking, are used to minimise some of the uncertainty; yet the mapping between the tracking data and the preprocessing algorithms is imperfect [41]. Further steps such as tissue segmentation, for example, of white and grey matter, background stripping and registration to a common atlas space, are important but difficult tasks for fMRI analysis [1, 49–51]. First, the modality is temporal with the potential for unaccounted motion between frames, which affects the quality of all three mentioned steps, e.g. for background stripping a single mask may not represent the whole sequence; second it contains three forms of matter—white matter, grey matter and cerebrospinal fluid—that have low contrast between them, hampering image segmentation and registration as both are reliant on contrast separations over the functional activity curve; and third, the anatomical structure is often fuzzy [1, 34, 46, 50, 51]. Moreover, each of the image processing steps require manual tuning and refinements, such as parameter selection, that can have a major impact on the data and introduce or compound multiple uncertainties about, for example, the accuracy of what data were removed or modified [13, 32, 52].

#### Other causes

Underpinning all these uncertainties are unknowns about individual patient physiology, including to what extent these physiological differences between patients affect the analysis outcomes [1, 12, 13, 35]. One of the major concerns regarding physiology is the effect of individual activity baselines in the brain. This refers to each person’s level and pattern of underlying brain activity that is related background brain function. This baseline is different for each person and can depend on other factors, such as time of day, functional state differences between scans and scanner environment [41], for a single patient at different time points [35]. This introduces uncertainties, such as whether the same image processing pipeline can be applied to different patients, and what it needs to be adjusted per patient.

### Image analysis

**Fig. 2:**
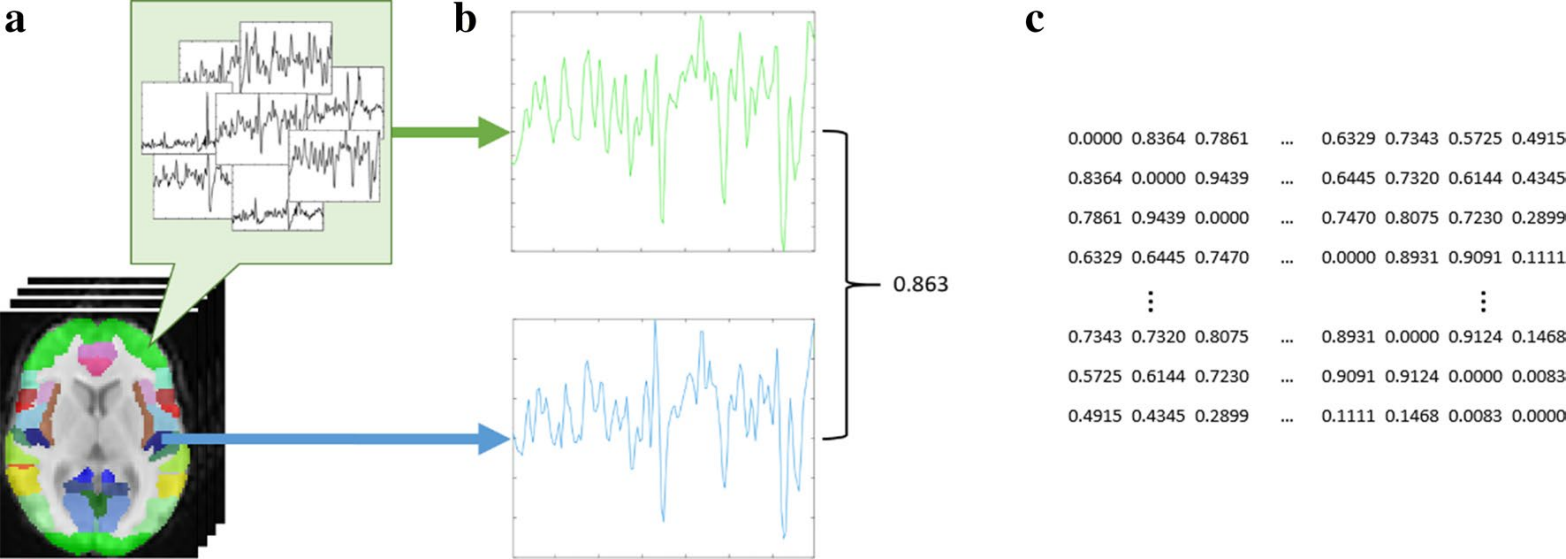
Functional connectivity networks are created by grouping voxels in the brain using parcellations: **a** voxels are grouped by anatomical regions from a predefined parcellation; **b** all the temporal signals in a region are summarised into a representative signal, and then the coactivation is calculated for each pair of regions; and **c** the coactivation values are formatted into a matrix that can be viewed as a network for analysis and interpretation. Uncertainties arise from this process regarding how well the parcellations fit each subject, heterogeneity in the temporal and spatial data, as well as the loss of spatial information in formatting as a matrix

Common image analysis approaches aim to abstract the data by, e.g. grouping related voxels by their temporal sequences into a single value representing the groups. This process is done to reduce the amount of data that requires analysis, making the process manageable and potentially reducing the impact of voxels affected by concerns such as noise, because they become a small part of the representative data point, rather than the whole data point [53, 54]. Image analysis can also provide the ability to directly compare images, which allows researchers to find similarities and differences between individuals and populations, for example, understanding Alzheimer’s disease [8]. Methods for doing this include voxel-based, such as PCA/ICA, which group voxels based on temporal signal, and node-based functional connectivity analysis, which groups voxels based on spatial location from predefined ROIs in a parcellation, as shown in Fig. 2. A comprehensive review of image analysis methods is beyond the scope of this paper, and interested readers are directed to recent works, e.g. [39, 50, 51]. These methods involve the selection of parameters and models, such as whether to delete time points prior to voxel-based and which parcellation to use in node-based analysis. However, these decisions cause uncertainty as there is no way to easily understand the impact of the decision on the data. For example, it is difficult to measure how well the parcellations in node-based analysis fit individual subjects. They may inaccurately map because when nodes are defined at the group level in standard space, there is a high risk for incorrectly defined node boundaries resulting from misalignment of individual subject data to the parcellation [55, 56]. Mapping can alternatively be done at the group level, which includes a step to refine the node boundaries in a way that is optimised for each individual subject. This improves the correspondence while still allowing some flexibility in specific node boundaries; however, the difficulties in measuring the fit of the parcellations have a large impact [55–58]. These uncertainties are in part due to the potential issues from the image acquisition and processing phase and thus compound the overall effects in the pipeline. The continued development of parcellations that attempt to provide more subject- and purpose-specific mappings of the brain, highlights this problem. For example, the authors of [56] note that their work is only an initial outcome of an ongoing development which will improve as better data and processes become available, both of which are impacted by the uncertainties in the image acquisition and processing phase.

Beyond selecting the parameters, other concerns arise in the steps to quantify how “coactive” regions of the brain are. Coactive refers to how similar the activity is over the temporal dimension for voxels or regions of the brain and is indicated by a single number, typically the correlation, for the temporal similarity of each pair of regions [39, 50]. This involves taking the temporal activity curve of each voxel in a region to create a curve that is deemed representative for the whole region, commonly through averaging the signals. The resulting abstraction replaces the hundreds of thousands of voxels, each with a unique activity curve, with a set of coactivation values for under ten to a few thousand ROIs. Comparison of regions and subjects, e.g. for evaluating diseases, is accordingly made much simpler, faster and more understandable. However, the steps create two forms of heterogeneity that lead to uncertainty: spatial and temporal. Spatial heterogeneity is more prevalent in node-based functional connectivity analysis as ROIs are based primarily on anatomical location and not the activity curves, although it is still a concern for voxel-based analysis as the grouping can be influenced by outlier voxels. Due to inherent limitations in parcellation, the resulting regions almost always contain a mixture of heterogeneous voxels that are not accurately captured by the representative signal, or in the case of larger outliers, affect the activity curve of the representative signal. Spatial uncertainty is compounded because each region is compared to other regions in the brain to measure the coactivity, thereby multiplying errors. Consequently, spatial heterogeneity leads to issues in the representative temporal signal regarding how accurately it summarises the activity in the region, and whether the heterogeneous signals are, e.g. noise [59]. Temporal uncertainty arises because brain activity can represent a number of state changes within a scan, such as short bursts of activity [60]. However, in abstracting the data, each pair of temporal signals is summarised into a single coactivation value. This value cannot reflect all the temporal variability within the data and thus may not be an accurate quantification of the coactivity during part of the sequence. Some statistical techniques, such as dynamic node-based functional connectivity analysis, which uses a sliding-window approach to summarise the temporal activity [61], attempt to minimise the temporal heterogeneity by accounting for the state changes within each window. However, these techniques still reduce the variability to a single number. The combination of temporal and spatial heterogeneity results in local and global levels of uncertainty. That is, local refers to how accurately the representative temporal signal depicts the signals of each internal voxel; and global is how well the relationship between the representative temporal signals is quantified in the coactivation value [30].

Finally, before progressing to the visualisation and human interpretation phase, the abstracted data are commonly processed further through thresholding [34]. In the case of node-based functional connectivity analysis, the threshold is used to filter coactivation values, so only those that are desired, e.g. highly coactive, are retained. Meanwhile, in PCA/ICA, the thresholding is performed at both a voxel- and cluster-level and in fALFF thresholding is performed based on the source signal details, such as frequency and amplitude. The voxel-level threshold is set as a cut-off parameter that determines which voxels belong to which group by quantifying how similar the voxel’s signal needs to be to the representative signal of the group. The group-level is then thresholded to filter out components that do not represent a high enough portion of the activity in the fMRI. Thresholding, however, creates new uncertainty as there are no accepted methods for defining a good threshold [34, 62–64]. Instead, thresholds that are simply considered “high enough” or “low enough” are commonly used, resulting in multiple questions regarding whether meaningful data have been thresholded out [62, 63]. This negatively impacts the reproducibility of studies and has led to a repository for sharing processed but unthresholded fMRI data, as each parameter and algorithm selected in the fMRI analysis pipeline to this step changes the data in a different way [65]; however, use of this repository is currently limited. Unlike the uncertainties inherent to the data acquired from the hardware, many of these issues have the potential to be improved through user interaction, visualisation and data analysis, as discussed in the following sections.

### Visualisation and human interpretation

#### Visualisation

Visualisation and visual analytics is designed to draw the attention of a user to certain aspects of the data over others. While this reduces the cognitive load on users and is done to highlight important aspects of the data, this inherently adds the risk of introducing uncertainty. For example, a visualisation may highlight a difference between coactivation values of two images; however, to do so, it is forced to make assumptions about which aspects are important, such as whether the absolute or relative coactivation differences are more important. This introduces uncertainties related to whether the, typically automatic, assumptions about what to highlight and what to de-emphasise are correct. These can then compound previous concerns, e.g. by emphasising coactivation between two regions that are affected by high spatial heterogeneity, as described in Sect. 2.2. This has a large impact because the coactivation does not carry any information about the heterogeneity and the coactivation will be read as meaningful when it is caused by issues in the data.

#### Cognition

Interpretation of complex fMRI data, even with the use of visualisations, is known to increase cognitive load that can lead to difficulty in understanding results. As an example, users can have trouble mentally reconstructing the brain due to the complex 3D structure with varying ROIs depending on the parcellation or known network [11]. Similarly, fMRI interpretation is heavily influenced by user expectation and bias, such as expectation of a certain coactivation between ROIs [66, 67]. This is exacerbated due to the current limited knowledge of the brain, which results in researchers having different ideas about what causes phenomena [66, 67]. Each of these can lead to misinterpretation of the fMRI data that has a compound effect as the interpretations are used to inform and create future studies [11, 66].

## Methodology

The visualisation and visual analytics tools described in the following sections were identified through searching leading publishers and major indexing databases: IEEE, Springer, Science Direct, ACM, Wiley and Google Scholar. The search terms: “functional magnetic resonance imaging”; “fMRI”; “connectome”; “functional connectivity”; “brain connectivity”; “neuroimaging”; “brain activity”; “neural activity”; “blood oxygen level dependent”; and “BOLD”; were used in combination with “visualisation”; “visual analytics”; and “information visualisation”; and the terms “uncertainty”; “issues”; “challenges”; and “concerns”. References lists of selected papers were also checked. As papers were found, we categorised them into the three phases described in Sect. 2 and then into themes based on how they presented fMRI data. We also performed specific searches on aspects such as temporal uncertainty or thresholding as we determined categories and themes; these augment our main search strategy. As a result, the works presented are not exhaustive; however, they cover in detail the key themes which need to be understood for future fMRI-UVA.

## Existing analysis processes, visualisations and visual analytics

Visual analytics tools aim to reduce the cognitive load on users during interpretation, intuitively present results of interpretation for communication between researchers, and allow researchers to interactively refine to present relevant information. Recently, there have been attempts at fMRI-UVA in the image analysis phase, exposing some of the uncertainties in the intermediate steps; these allow for decisions to be made about aspects of the data quality, for example, temporal heterogeneity as introduced in Sect. 2.2. Yet, many of the processes in the fMRI pipeline utilise statistical toolkits that introduce the uncertainties without employing any fMRI-UVA. In this section, we discuss the key toolkits, processes and software libraries that are used throughout the pipeline. While our analysis focuses on the visualisation and visual analytics tools, we also introduce standard statistical methods where these are commonplace.

### Image acquisition and processing

**Fig. 3:**
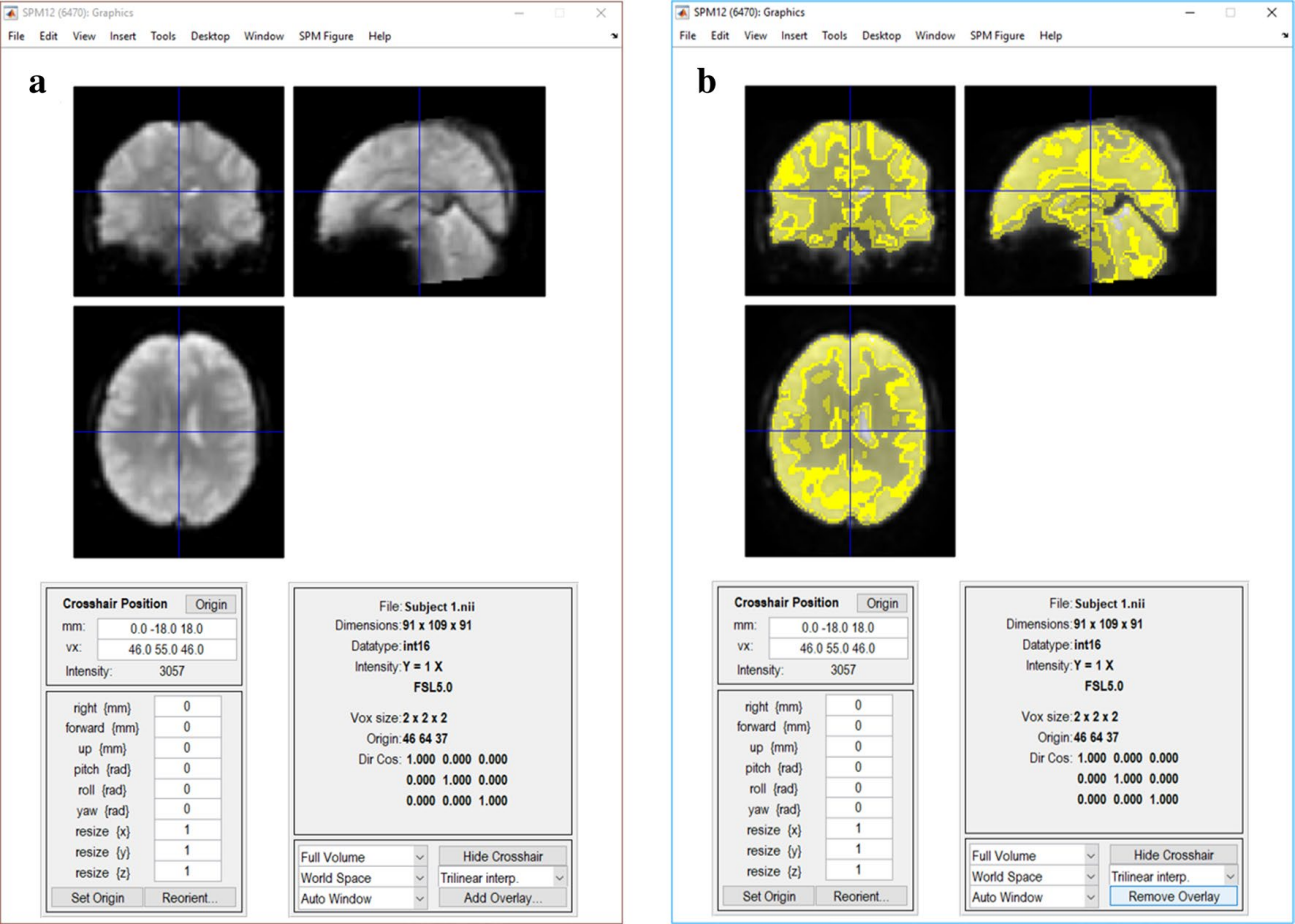
An example of an fMRI visualisation using SPM [71] showing the results of white matter (WM), grey matter (GM) and cerebrospinal fluid (CSF) after segmentation with FSL FAST [70]: **a** the original fMRI image with a voxel size of 2 × 2 × 2 mm; **b** the image with the uncertain, calculated matter types overlaid in shades of yellow (WM in the dark yellow; GM in the middle yellow; CSF in the bright yellow)

To the best of our knowledge, no research exists specifically for fMRI-UVA of the image acquisition and processing phase, largely due to the recent emergence and relative immaturity of fMRI-UVA in comparison with the range of established statistical toolkits. Standard statistical toolkits, such as AFNI [68], Freesurfer [69], FSL [70] and SPM [71], which perform processes introduced in Sects. 2.1.1 and 2.1.2, are commonly used. Often, these processes contain semi-automatic steps that allow a user to visualise the data they have processed and make decisions regarding the parameters. For example, Fig. 3 presents visualisations available in the SPM processing software [71] that can be used to adjust parameters relating to segmenting white matter, grey matter and cerebrospinal fluid. However, selecting these parameters is a difficult, error prone task [32]. Consequently, it can be very challenging to estimate the effects of parameter selections before they are performed, while observing whether a selection has caused an issue relies on extensive visual inspection as the tools do not currently present adequate guidance [1, 12, 13, 35].

Despite the lack of existing fMRI-UVA in this phase, there are visualisation tools from other medical imaging domains that could be useful for the issues in image acquisition and processing. For example, volume rendering of segmentation uncertainty, e.g. highlighting areas that are deemed to be “suspicious” [22], could allow an expert user to evaluate how well a skull stripping process has been performed. However, visualisation analytics for data acquisition issues, such as motion, noise, bias field and patient biology, are not, to our knowledge, studied in other imaging domains due to the robustness of existing procedures for the modalities. Yet, for fMRI there is a need to discover more about the effects of the uncertainties as an adequate level of robustness has not been reached [13]. For these concerns, researchers can look to UVA tools from outside the medical imaging domain. For example, TimeTubes [72] is a visual analytics tool that highlights the uncertainty in astronomical observations of “blazars”, which relate to black holes. The data similarly contain 4D spatial and temporal information, have to be reconstructed from signals with multiple artefacts and can vary depending on the source black hole, similar to patient physiology. TimeTubes uses opacity, shape, size and interactivity in its visualisation to indicate potentially inaccurate or missing information. The techniques explored in the tool, and other similar tools made for complex 4D data, could be adapted to fMRI-UVA. For instance, the TimeTubes technique could be adapted to map physiological measures during the scan, such as heart rate, which may assist in understanding the patient’s biology.

### Image analysis

**Fig. 4:**
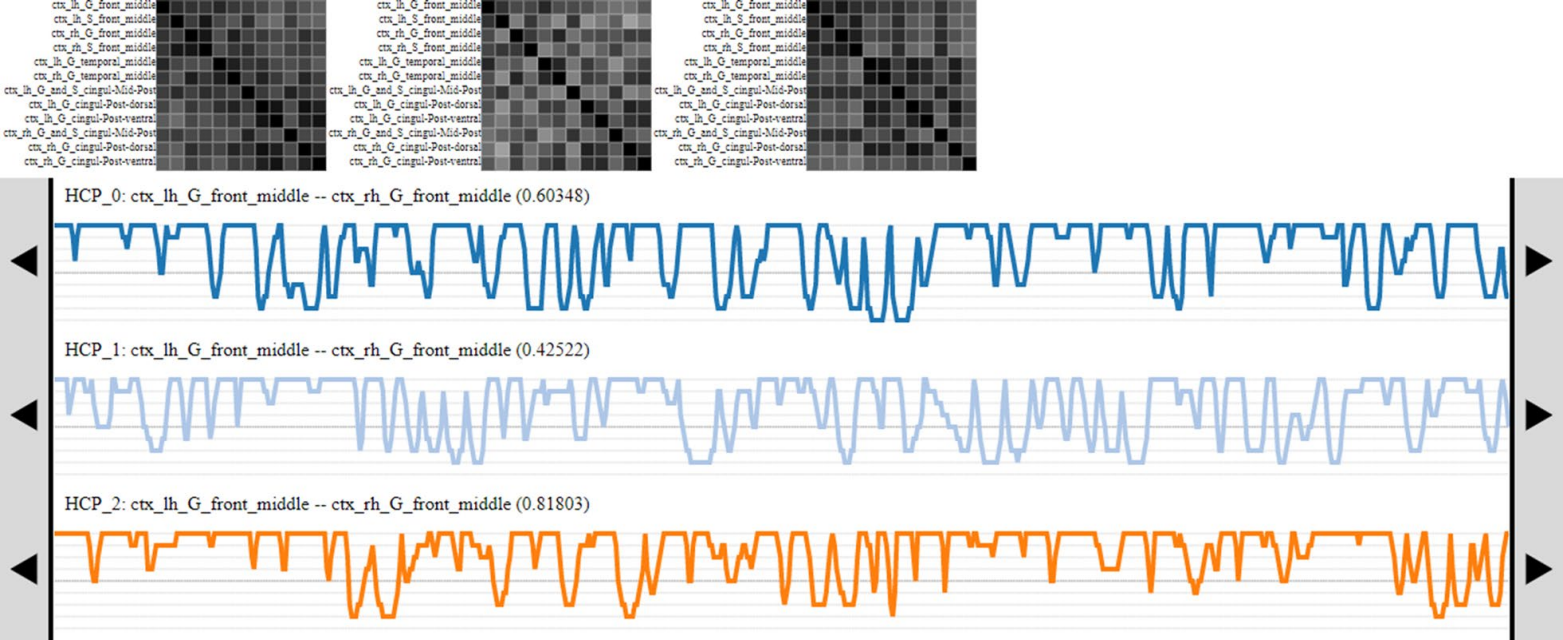
The TemporalTracks interface. The FCN matrices at the top change to reflect the user modifications to the tracks below. Each track shows the pairwise coactivation beween two ROIs—when lines are at the top, the ROIs highly coactive and when the lines are at the bottom, the ROIs are not very coactive
- TimeCurves [74], in Fig. 5, was not designed specifically for fMRI-UVA of temporal sequences; however, it is useful in examining global uncertainty. The tool can be used to visualise cycles, repeated patterns or rapid changes in the overall signal in a temporal sequence. In contrast to TemporalTracks, TimeCurves is applied to whole fMRI images, rather than ROIs. The visualisation takes the linear temporal sequences of all ROIs and visually bends the timeline based on how similar temporal points are. Thus, features such as cycles can be used to draw out subsets of time for detailed uncertainty analysis, e.g. repeated machine artefacts. Similarly, large jumps in the curved timeline may be indicative of outliers and other features, such as temporal state changes, which can negatively impact further interpretation. The fMRI-UVA tool provides interaction for changing the linearity of the overall line, so a largely clustered graph can be slowly unpacked.

Most of the limited fMRI-UVA research into the image analysis phase has targeted the temporal aspects of the data because the temporal aspects are generally what is used to compare patients during analysis. Such tools can be used to determine temporal periods that may contain differences to the whole sequence, or be indicative of uncertainties in the temporal sequence. This can be done for local or global uncertainty, wherein local allows users to observe uncertainties between individual ROIs, while global allows users to observe uncertainties across the whole brain. Notable fMRI-UVA tools for temporal analysis include:

- TemporalTracks [73], shown in Fig. 4, was designed to explore how well node-based functional connectivity values represent the underlying brain activity of ROIs for local uncertainty and whether subsets of time hold important information, such as unique patterns. Interaction with TemporalTracks is provided, so users can compare the temporal sequences between pairs of ROIs to determine whether the coactivation value is accurate or whether it is affected by any underlying differences, such as outliers caused by unaccounted head motion or a machine artefact. Users can also shift temporal sequences and view how the FCN changes during subsets of time which can assist with uncovering phase-based concerns. This visual analytics tool was developed specifically to target the temporal uncertainty prior to human interpretation of the FCN information.

**Fig. 5:**
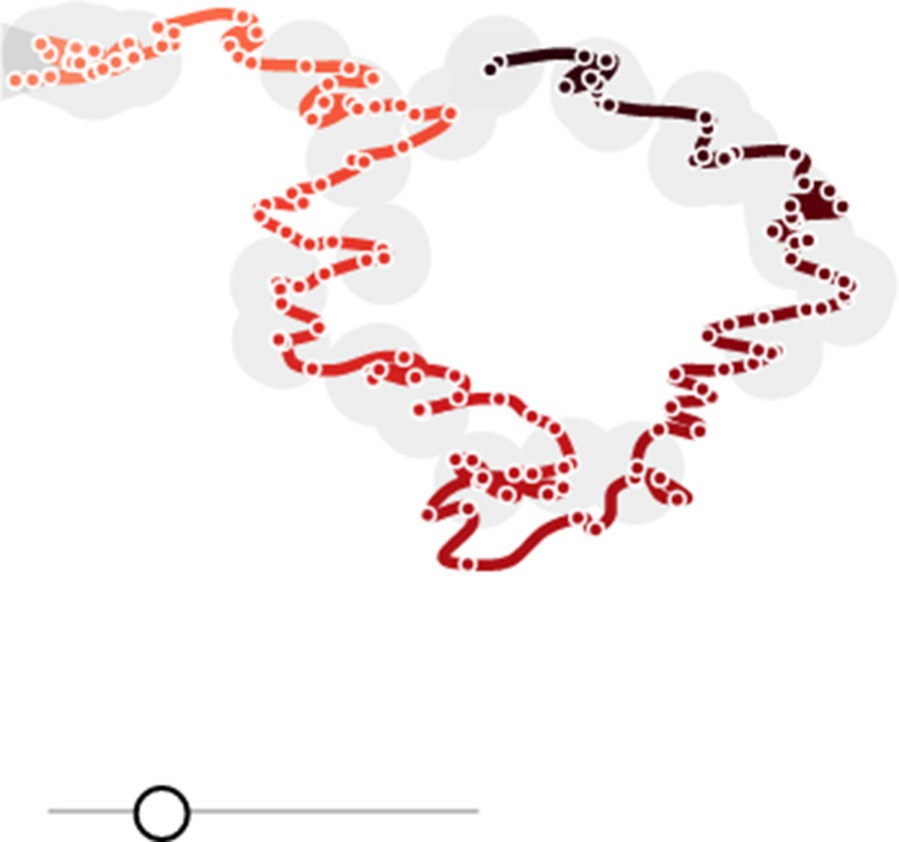
An example of a TimeCurve. The location of points on the plane is influenced by the underlying temporal measures. Cycles represent repeating patterns, in which close points or tightly clustered sections are highly similar. The bar at the bottom adjusts the level of curvature presented
- SmallMultipiles [28], in Fig. 6, was proposed for visualising “fuzzy state changes” in brain activity over time by grouping different brain activity states. Thus, it was designed to uncover global uncertainty in the temporal data. In the visualisation, temporal points are represented as FCN matrices for the whole brain. These matrices are then clustered into “piles” which represent different brain states. Therefore, users can observe piles which have potentially meaningful or uncertain data, e.g. coactivation outliers, to determine whether the brain state is useful for further human interpretation or whether it is the result of an issue, such as head motion. Interaction is provided in the fMRI-UVA to alter the level of clustering and to flip through the piles, allowing for drill-down analysis.

**Fig. 6:**
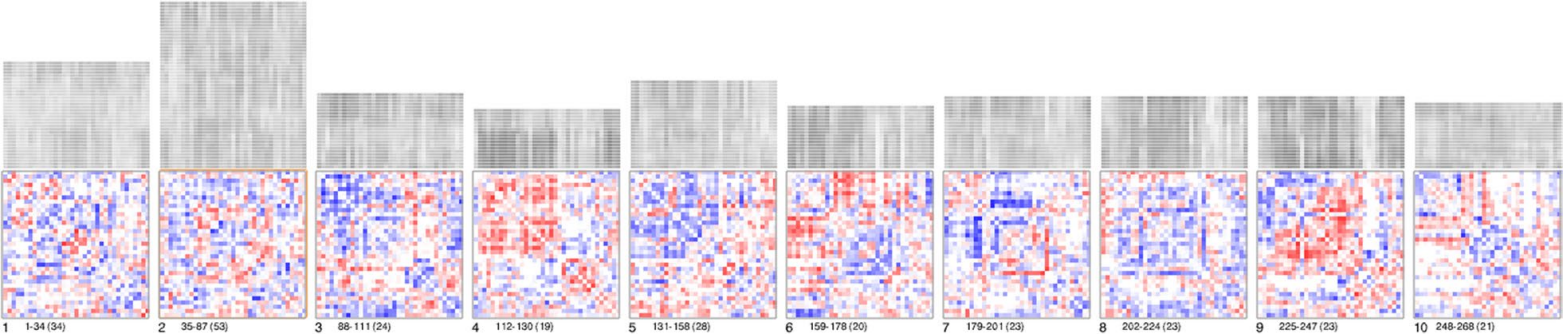
Example of SmallMultipiles. Each pile consists of highly similar frames in the temporal sequence. Users can adjust the number of piles (level of clustering). The height of piles can be used to see how common patterns are in the temporal sequence

The major concern of threshold selection, as discussed in Sect. 2.2, has not, to our knowledge, been addressed in any visual analytics studies. However, as selected thresholds change the network structure in node-based analysis, visualisations that summarise the overall network structure for comparison may be useful. For example, graph decomposition techniques are used to simplify large networks before they are visualised. This could be used to show an overview of different threshold values or techniques, allowing for direct comparison of the thresholds and indication of when different networks undergo similar changes. Such an approach may lead to simpler selection of patient-specific thresholds. For other uncertainties in the image analysis phase, such as the fit of atlases and known networks, spatial heterogeneity, biological differences between patients, and the compound effects of the image acquisition and processing phase, there are many UVA tools that can be adapted. For example, spatial heterogeneity could be represented in a similar visualisation to what was presented in [75], which used colour and associated graphs to visualise heterogeneity for classification. Such techniques could be combined with statistical approaches for improving the fit of atlases, e.g. [57, 58, 76], by indicating visually what impact different parameters have on the heterogeneity of ROIs.

### Visualisation and human interpretation

**Fig. 7:**
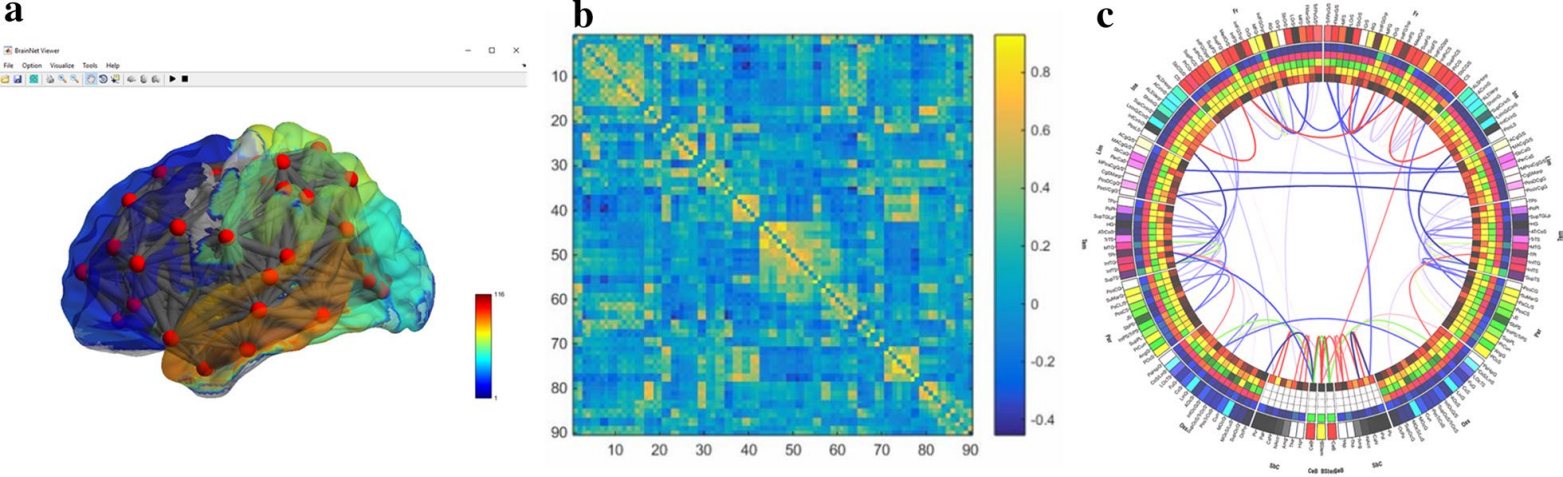
Examples of the three most common direct connectivity measure visualisation techniques: **a** node–link diagram which is overlaid on a 3D anatomy (created in BrainNet Viewer [77]). This minimises the need for mental reconstruction of the anatomy, but can increase clutter and imply direct connections; **b** matrix visualisation where pairwise coactivation values are displayed on the grid. This requires more mental reconstruction of the anatomy, but it is better for subject comparison and is clutter free; **c** radial connectivity diagram (created in the pipeline by Irimia et al. [78]). This implies anatomical location through node ordering, while also not implying direct connections and minimising visual clutter
- *Anatomy*-*based visualisations*, which present fMRI data on surface and volume renderings of the brain. One of the seminal works in this category, MindSeer [81] (Fig. 8a), combines volume visualisation of the raw fMRI and structural MRI data, with a surface rendering of the brain with coloured highlights for connectivity data. Interaction is provided to navigate the scene, alter transparency and threshold the different data sources. This minimises the need to mentally reconstruct anatomical features and thus reduces potential misinterpretation. A more recent significant example, by Böttger et al. [82] (Fig. 8b), displays visual glyphs on a 3D surface rendering of the brain. The glyphs are designed to encode functional information between ROIs. This approach is designed to minimise cognitive load in mentally reconstructing the brain; however, it is susceptible to visual clutter and the edges, when shown, can seem like direct physical connections rather than, e.g. correlations. A third work, by Li et al. [83] (Fig. 8c) is a visualisation which encodes functional information in surface rendered textures. In this way, the tool is able to encode some of the underlying temporal information into surface renderings of ROIs, rather than simply presenting coactivation values. As a result, the visualisation has the ability to potentially display regions affected by heterogeneity and temporal variability as an unintended benefit alongside reducing the human uncertainties. Interaction is provided to navigate the 3D space and to threshold measures. Users can also view the ROI data in polar coordinate space, which partially overlaps with the next category.

While the first two phases of the pipeline had limited fMRI-UVA research, the visualisation and human interpretation phase has been addressed by many solutions due to the direct benefits of reducing cognitive load on users and need for interactivity during interpretation. Although fMRI interpretation can take many forms, these works principally focus on node-based functional connectivity analysis because of the wide use of the technique and because of the inherent, yet complex, network structure. These can be categorised into:

- *Direct connectivity measure visualisations,* such as matrices, node–link diagrams and radial connectivity diagrams for FCN analysis. One of the most widely used fMRI visual analytics tools, BrainNet Viewer [77] (Fig. 7a) is in this category. The tool combines node–link diagrams with a 3D rendering of the anatomy. The tool allows interaction for user navigation of the scene, filtering and thresholding. By doing so, uncertainty relating to the mental reconstruction of the anatomy is minimised; however, there are concerns with visual clutter and implied direct connections. A notable example of a radial visualisation by Irimia et al. [78] (Fig. 7c) uses node ordering to indicate anatomical location of ROIs. By doing so, the authors aim to reduce the impacts of cognitive load on interpretation as users have to undertake less mental work to map an ROI name to the spatial location of the ROI in the brain. Alongside, the tool also presents secondary measures, such as grey matter volume, in coloured boxes and circles. The tool is designed to sit statically at the end of a pipeline as a reporting tool and thus has limited interactivity. The techniques in this category are often used for comparison of subject graphs due to the nodes and edges always being in the same location, which helps to facilitate comparison and minimise cognitive load [79]. A user study by Alper et al. [80] evaluated alternative direct connectivity visualisations—various node–link and adjacency matrix visualisations—for their strength in comparison. They found that the matrix visualisations improved user accuracy and speed of performing tasks because they are better at presenting large volumes of dense data without visual clutter. Moreover, matrices were also preferred by the users.

**Fig. 8:**
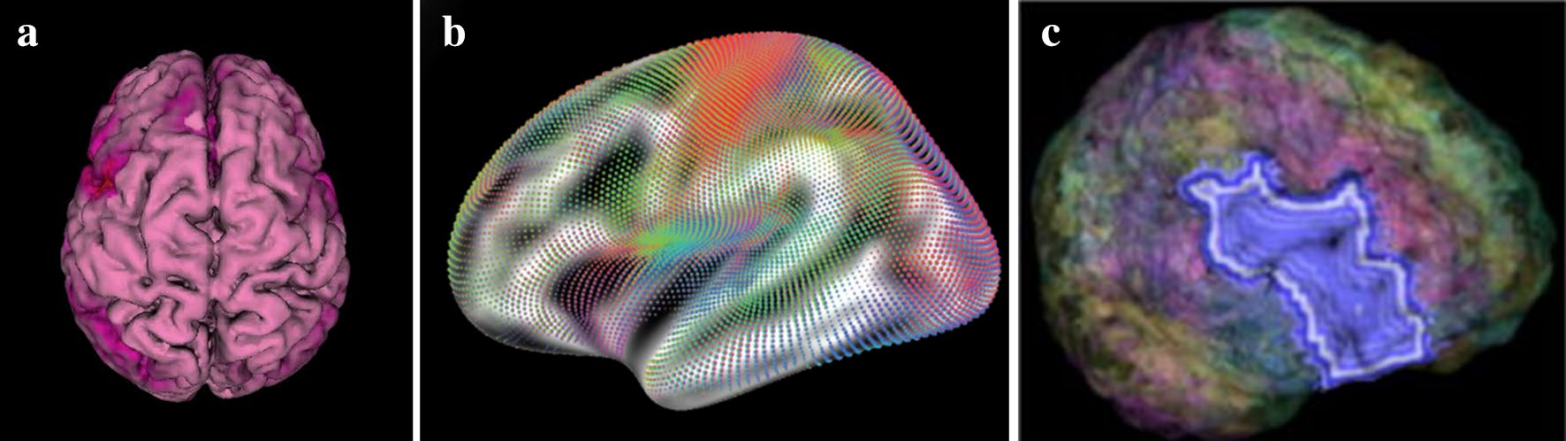
Examples of anatomy-based visualisations; these minimise the cognitive load required to map fMRI information back to the anatomy. **a** The connectivity data in a heatmap on the brain surface (created in MindSeer [81]). The software also allows for volume and raw fMRI visualisation in a cutaway fashion. **b** Glyph visualisation on a slightly inflated brain surface (Böttger et al. [82] via the NeuroBeuro [84]). This can present more information for interpretation without adding much extra human processing because it adds more depth and texture in the glyphs. **c** An extension to surface heatmaps (from Li et al. [83]) which encodes extra temporal information into the ROI surfaces. This information could be used to help understand underlying data uncertainties
- *Abstract anatomy visualisations* are an extension to anatomy-based visualisations which modify the surface and volume renderings to make them easier for humans to view and comprehend. The trade-off is that some mental reconstruction of the original anatomy is required and they are not as clutter free as direct FCN visualisations. An early example, called Brain Constellation [85] (Fig. 9a), flattens the anatomical data by performing PCA on the ROI positions and areas. FCN information for each subject is then presented in a small multiples display. Interaction for navigating the 2D spaces and filtering is provided. Such an approach minimises the cognitive load of understanding 3D data on a 2D screen; however, the diagrams imply direct connections between ROIs and some mental reconstruction is required to relate back to the 3D anatomy of the brain. PyCortex [86] (Fig. 9b) is one of the more widely used tools; it flattens and opens the 3D structure of the brain before highlighting important details using colours and visual cues including outlining. Users can interact with the visualisation by altering how flat the brain surface is and navigating the 3D space. Users can also change which measures are shown and how they are displayed. Alternative approaches provide interaction and visual variability to the abstract anatomy; instead presenting visual information in associated elements, such as glyphs. For example, Yang et al. [27] combined a 2D sketch of the brain with a NodeTrix visualisation of the functional data (Fig. 9c). This was done to facilitate comparison of subjects while balancing available information and the requirements of cognitive load in mentally reconstruct the anatomy. Users can view networks side by side and interact by filtering and thresholding. This representation also indirectly helps with visualising local area issues that may be caused by issues such as motion or machine artefacts as the brain is subdivided into sections for each trix. Thus, outliers in one section of the brain (e.g. in the case of accidental auditory stimulation during a scan which can cause activity in the somatosensory cortex [87]) may be visible.

**Fig. 9:**
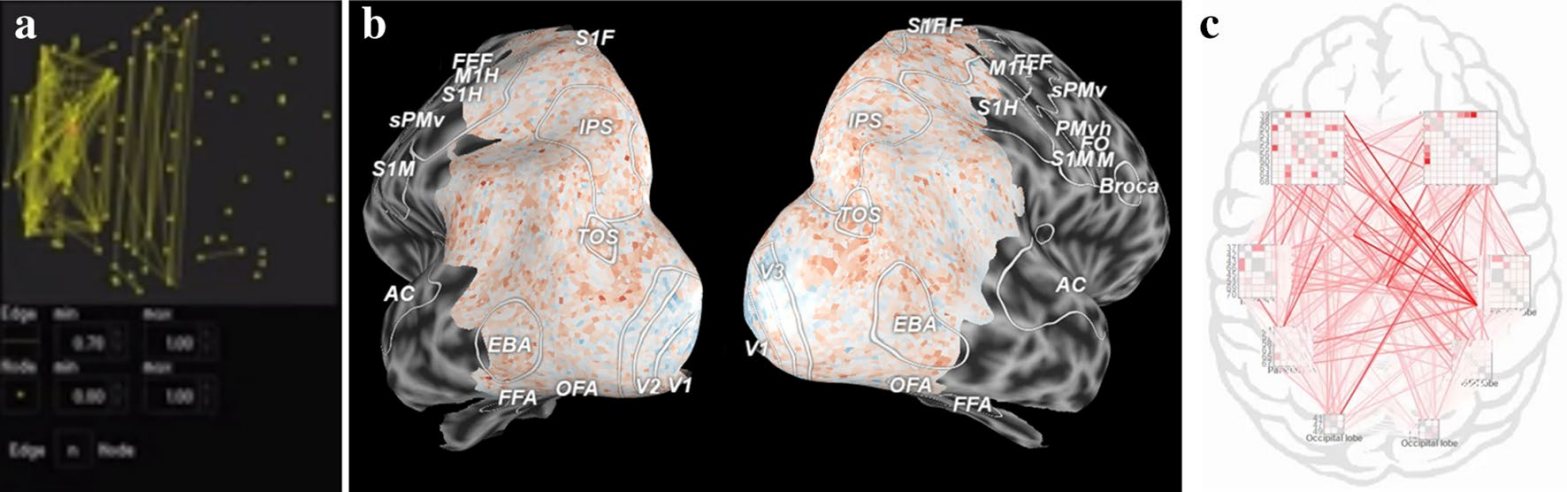
Example abstract anatomy visualisations; these trade off some of the benefits of the direct anatomical reference for improved representations of the fMRI data: **a** two-dimensional representation of the ROIs throughout a brain with edges displaying coactivity (from Brain Constellation [85]); **b** a split and flattened view of the brain surface with a heatmap overlaid (made in PyCortex [86]); **c** NodeTrix visualisation on a 2D sketch of the brain, showing connections and local neighbourhood information, which may be useful for investigating data uncertainties
- *Abstract visualisations* are ones which take the available data and present it in a way that does not obviously represent the anatomy or the underlying BOLD; from, e.g. FCN, PCA or voxel clustering. These techniques usually perform some extra processing on the data prior to displaying the visualisations. As a result, the works can be quite disparate; however, they have similar benefits for human uncertainty. One of the notable works, by DeYoe and Raut [88], creates what they term a functional field map for analysis of fMRI during a visual task. This combines the fMRI data with details about the visual location of the stimuli to create an orthogonal graph. Thus, both input sources can be represented without visualising the anatomy and comparison of the functional field maps can be performed with minimal visual clutter. Such an approach may be appropriate in specific circumstances, for example, when the anatomical location is constrained to the visual cortex, so mental reconstruction of the anatomical neighbourhood is not as critical. Other abstract visualisations focus more on the analysis of population data sets. A significant recent example, by Fujiwara et al. [89], projects subject FCN data into what is termed a multidimensional scaling space (Fig. 10). This allows subjects to be grouped and classified easily without users needing to remember specific FCN details. The tool also allows users to drill into the data by selecting and presenting FCN matrix visualisations and anatomical renderings of node–link diagrams. As a result, the fMRI-UVA tool minimises the amount of information users need to remember when performing high level comparisons. Thus, a large amount of misinterpretation and cognitive load concerns are accounted for.

**Fig. 10:**
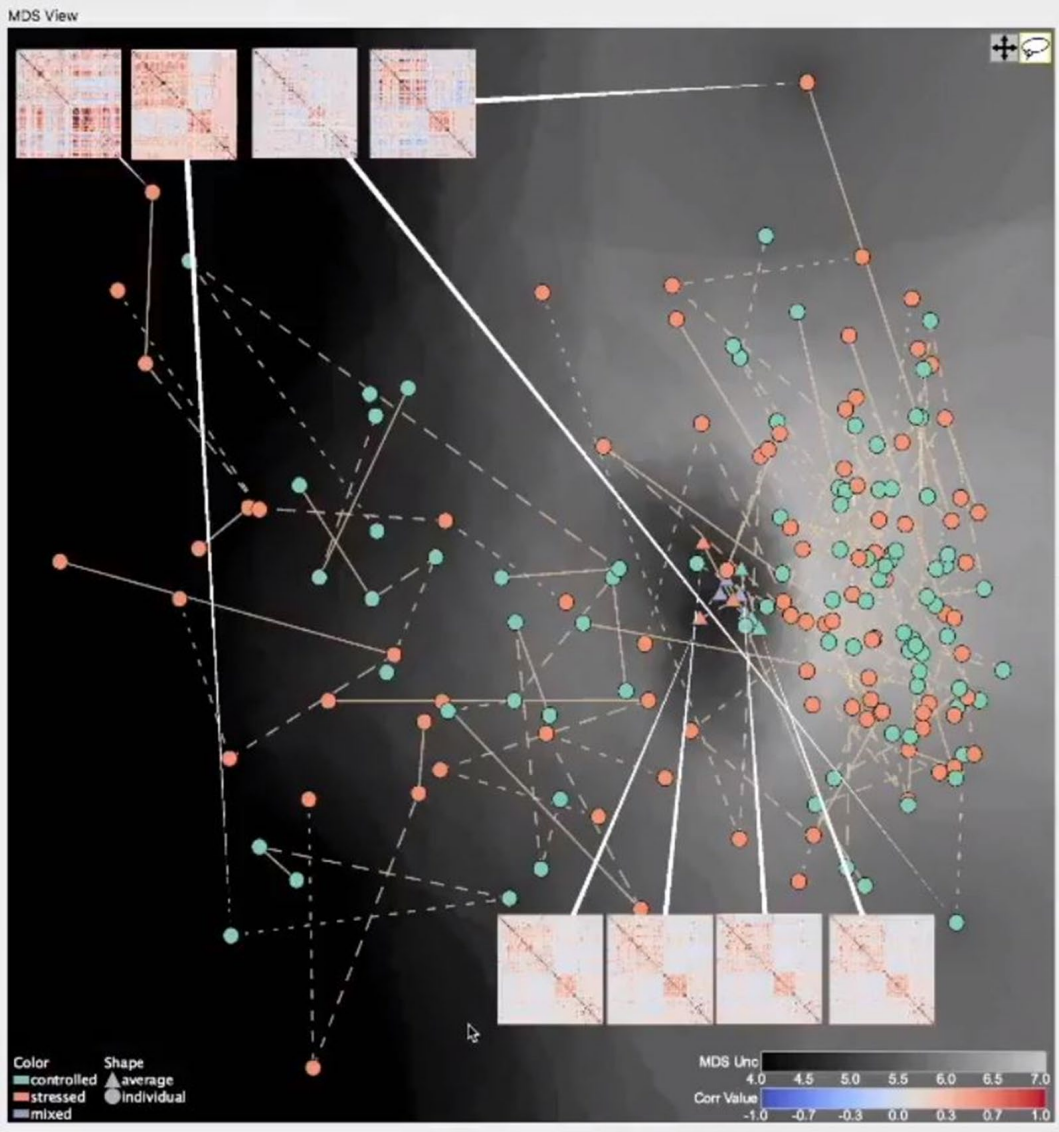
Example of an abstract visualisation (by Fujiwara et al. [89]). This visualisation allows users to explore population datasets without needing to remember specific details about subjects, thus reducing cognitive load. The drill-down functionality, shown in the matrices, means that users can access the information when required

**Fig. 11:**
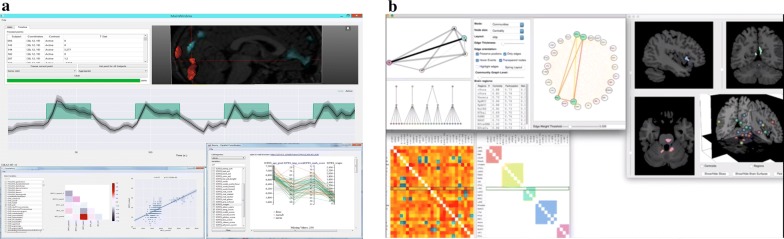
Examples of multimodality and data source windowed approaches: **a** three windows created in BraViz [90] showing an fMRI timeline above two windows of population demographic statistics; **b** Brain Modulyzer [92] displaying derived statistics alongside fMRI connectivity data
- *Linked views* similarly enable users to view fMRI data from multiple angles. However, the visual elements are fixed in size and position. One of the main examples for fMRI-UVA, CereVA [91] (Fig. 12), uses linked views to present fMRI data from different processing pipelines. This tool has traded off some of the flexibility of the windowed approaches for a structured interface containing defined module location, thus prioritising repeatability and minimising visual searching [79]. The modules are designed to present the FCN data in direct connectivity visualisation, alongside temporal and anatomical visual components. Interaction is provided so that navigation in one section is mirrored in the other visual components. Such an approach means there is more information for a user to interpret, yet the information can provide direct insights into the data uncertainties, such as heterogeneity and temporal artefacts. Moreover, the inclusion of the anatomy is designed to minimise cognitive load in mentally reconstructing the location of ROIs without implying direct connections or cluttering the FCN visualisation.

Each of the above works is primarily designed to encode the information on a single graph or in one graphical window—some of the tools do provide other windows in a drill-down fashion—which minimises the screen space required and allows users to understand all the information at once. Other approaches, e.g. [90, 91], use a windowed approach which allows the user to present multiple graphs about related information from the fMRI and from other data sources, such as diffusion tensor imaging (DTI) or electroencephalography (EEG) readings, which can provide more information about the subject and add to the interpretation. Such tools can be categorised into:

- *Windowed approaches*, which present data in multiple movable and resizable windows. These often augment the fMRI human interpretation process by including data from other imaging modalities, e.g. DTI, and data sources, e.g. EEG. A notable example is BraViz [90] (Fig. 11a) in which users can view fMRI data alongside DTI, underlying temporal and second-order statistics. The benefit of presenting these disparate data types in windows is that users can gather more information about the brain, yet they are only presented with what their interested in. This can help in preventing cognitive overload; however, because the windows can change size and move, the interface itself can lead to issues caused by clutter and visual searching [79]. This approach is also used to present fMRI data from multiple angles, e.g. Brain Modulyzer [92] (Fig. 11b). The provided interaction similarly enables exploration of the different windows as they are linked together and users do not have to mentally couple the disparate data sources. However, the interface is again not structured, which leads to clutter and searching issues.

**Fig. 12:**
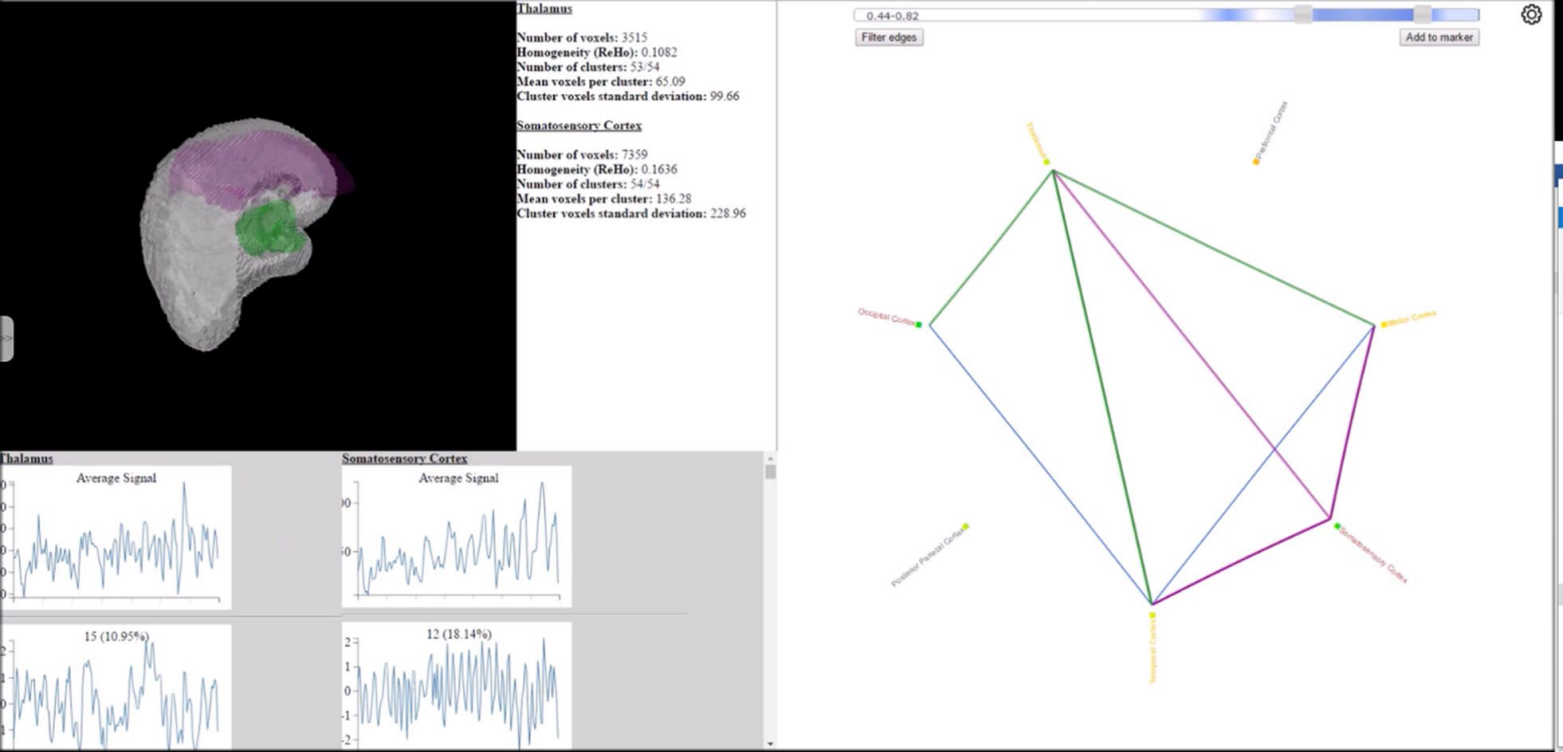
Example of viewing fMRI from multiple angles in CereVA [91]. The three linked views are statically placed, so users always know where to look, minimising visual searching. Each of the elements also presents a different view of the underlying fMRI data, which is designed to assist with uncovering the data uncertainties alongside minimising human issues, such as cognitive load

Many fMRI-UVA tools which are designed to target human uncertainties are widely used and thus present a platform and common set of visualisation techniques that could expedite research into further fMRI-UVA. Specifically, most of the presented works focus on reducing certain aspects of cognitive load, such as mental reconstruction of anatomical location; these benefits could be combined with visualisations which target data uncertainties, as demonstrated in CereVA [91]. When selecting a category of fMRI-UVA tools, users should consider the purpose of their study alongside the expertise of the users. For example, direct connectivity visualisations are well suited to comparing small numbers of subjects, while abstract visualisations may be better for larger populations. Similarly, anatomy-based visualisations are likely better suited to novice users who do not know the structure of the brain well. The various windowed approaches can also be useful depending on the study as they can facilitate multiple data types for studies such as [93–95].

## Research opportunities

There are several research opportunities to be explored in the field of fMRI-UVA which we group into two rough, interrelated streams: summative and targeted approaches. Summative approaches refer to works which aim to present the effects of the uncertainties alongside the data for interpretation; the goal is to present information about the uncertainties such that the interpretations can be made with the potential issues in mind. In contrast, targeted approaches refer to works that aim to resolve or minimise the impact of specific issues during the fMRI analysis pipeline. This often means semi-automatic tools to be used during individual processing steps, such as for misrepresentation of the temporal variability, or during skull stripping.

### Summative approach

Although current fMRI-UVA approaches for the visualisation and human interpretation phase regularly present multiple different graph representations of the fMRI and associated data types, e.g. [77, 90], they often fail to convey the uncertainties in the underlying data and compounded uncertainties arising from earlier, mandatory, pipeline phases. These can have a huge impact on the outcomes of human interpretation [13] and can further embed user expectations or bias [66, 67]. Thus, summative approaches need to be developed to present these uncertainties during the human interpretation and visualisation phase, so they simultaneously allow for interpretation of the data, while providing an understanding of the impacts from all the pipeline phases. The key benefit of this approach is that users can improve and adjust their interpretation based on the quality of the data [17, 18]. For example, displaying the heterogeneity of ROIs in node-based functional connectivity analysis will have a huge impact on the interpretations by allowing a user to judge the quality of the coactivations. Research should attempt to explain and summarise which image processing pipelines have been utilised, and how these may impact the fMRI information available for interpretation. Furthermore, frameworks should be cognizant that the abstractions themselves are imprecise and show potentially spurious measures. Interactivity must be embedded that allows a user to observe these potentially spurious measures and then form and test hypotheses related to the data concerns that may have formed them. Likewise, instead of presenting the results of the analysis pipeline for interpretation, summative solutions should be made to follow a top-down approach that first presents an overview of the data, the abstraction and the uncertainties, then allows a user to drill down and adjust parameters along the whole analysis pipeline. The crucial challenge of the summative approach is to present such a large volume of information, with a high proportion of it being uncertainties, in a way that still minimises cognitive load and increases human understanding.

Consequently, future work in this area is about taking steps to create a single fMRI-UVA entity. One of the initial steps should be in discovering the interfaces between the existing visualisation techniques and the presentation of the underlying uncertainties. Current statistical approaches are taking this interconnected approach, combining multiple modalities or statistical analysis methods together, which allow for a deeper understanding of the brain as an overall system [96, 97]. Extending on this idea by visualising the outcomes of different techniques together holds great promise. Similarly, using one technique to influence the interpretation of another will be able to improve analysis. For example, the voxel-based PCA/ICA could be used in fMRI-UVA with dynamic node-based functional connectivity to weight or visually highlight the contribution of each temporal sub-sequence. The possibility for this approach is highlighted by recent tools, such as BraViz [90], beginning to integrate multiple data types into their interfaces. With some further research into creating such multifaceted approached, the balance between information and cognitive load can be found.

The next step to be taken in realising the summative approach is to integrate the temporal, spatial and abstraction contexts into a single interface and then to display the uncertainties present in each context, thereby being able to highlight the relationship to the pipeline and between each of the contexts. Such an approach has recently become possible due to the combination of statistics, such as ReHo [98] for calculating the heterogeneity of regions, with the image analysis fMRI-UVA tools discussed in Sect. 4.2.

### Targeted approach

Targeted fMRI-UVA has the potential to be used to minimise or remove some of the uncertainties in the fMRI pipeline. This is a process that has been used to great effect in other medical imaging fields, e.g. quantifying the differences between fibres in diffusion tensor imaging [99]. However, due to the temporal aspect of fMRI it is challenging to isolate meaningful measures at specific frames or subsets for visualisation. Moreover, other issues, such as the poor SNR and low resolution, hinder direct visualisation. Overcoming these barriers is possible and should begin with building upon existing UVA tools from other these medical imaging fields to target how the imaging uncertainties manifest in fMRI. Examples from the other medical imaging fields commonly use opacity, colour, shape and area as modifications on visualisations to compare effects of unknowns [23]. Visualisations for other data related to the brain will be a good place to start; for example, temporal neuron and EEG data, e.g. [100, 101], for exploring more into temporal uncertainties. In adapting these tools to fMRI, there will be missing information; but if data are unknown, it should be either presented in a raw form, possibly alongside a heuristic visualisation, or noted as such. This will increase cognitive load on users; however, it will also improve the outcomes of the fMRI pipeline, which still lands it on the positive side of the trade-off. Targeted methods should also be cognizant of which phase they are designed for in the fMRI pipeline and accordingly take input and create output in standard formats. This will allow them to be integrated, like the summative tools, into standardised software.

Future work into targeted approaches should be more diverse than for summative visualisations. As with the summative approach, uncertainty visual analytics research into analogous fields, such as other medical imaging domains, or fields that deal with complex 4D data, should be exploited. Similarly, more statistical methods can be combined with interactive visual tools, as current fMRI-UVA is heavily tied to FCN analysis alone. Suggested future works include:

1. An initial key area that should be targeted is fMRI-UVA of the temporal sequence. As discussed in Sect. 4.2, researchers are beginning to create tools for temporal uncertainties, while statistical research into the temporal data is creating techniques, such as dynamic node-based functional connectivity approach [61]. As a result, the processes surrounding temporal uncertainty in the data are reaching a level of maturity that benefits the emergence of visualisations.
2. Correspondingly, there has been recent research that attempts to adjust parcellations to better fit individual subjects, e.g. [57]. The combination of such techniques with interactive visualisation enables targeted fMRI-UVA that was not previously possible.
3. Reducing spatial heterogeneity is another target area which can benefit from statistical methods, such as ReHo [98]. Approaches can be combined with the previous application of parcellations, or they could be designed to sit earlier in the pipeline during the image acquisition and processing phase.
4. Thresholding concerns can benefit from graph topology and summarisation methods, e.g. graph decomposition [102], as these allow for comparison in small amounts of screen space.
5. Application to other processing methods, such as PCA/ICA, can use works already created for fMRI-UVA, such as volume rendering to assist with parameter selection and to elucidate potential issues in independent components analysis.

Targeted fMRI-UVA approaches should limit automatic statistical processing to those that are known to be accepted, or otherwise present the risks that come with the statistical analysis. These should aim to provide an ordinal, interval or ratio comparison, while highlighting where the uncertainties are. In this way, targeted solutions will be positioned to integrate into the analysis pipeline and help in uncovering the concerns surrounding fMRI.

### Implications

Both summative and targeted approaches to fMRI-UVA have the potential to greatly impact the future of brain research. While all the suggested methods will increase the cognitive load on users compared to existing approaches, due to the increase in information presented and the complexity of the information, the overall benefits of presenting uncertainty information far outweigh the drawbacks of increased cognitive load. Further, as the fMRI-UVA solutions are developed, researchers will discover how they can present the information without increasing the cognitive load too much. Similarly, some of the suggested methods have not been attempted because they are challenging with fMRI data. Nonetheless, we believe that fMRI research has recently advanced to a point where the uncertainties are causing major impacts on published findings that must be resolved. Moreover, these impacts allow researchers to better understand what the uncertainties are and how they compound, thus creating a large knowledge based and in turn reducing the challenges in creating fMRI-UVA.
